# Heat shock protein response during fixed intensity and self-paced exercise in the heat in young, healthy women on oral contraceptives compared with young healthy men

**DOI:** 10.17159/2078-516X/2022/v34i1a11757

**Published:** 2022-01-01

**Authors:** K J Onus, J Cannon, F E Marino

**Affiliations:** School of Allied Health, Exercise and Sport Sciences, Charles Sturt University, Panorama Ave, Bathurst, NSW 2795, Australia

**Keywords:** cellular stress, females, Hsp, performance, thermoregulation

## Abstract

**Background:**

Heat shock proteins respond to a variety of physiological and environmental stresses, including heat stress, ischemia and endotoxic shock. Hormonal changes during the female menstrual cycle can have a thermogenic effect on body temperature. The monophasic oral contraceptive (OC) pill provides low doses of progesterone and oestrogen over the course of the normal menstrual phase. There is little evidence regarding the combined effects of OC on exercise performance and heat stress with respect to heat shock protein response.

**Objectives:**

This study aimed to determine the response of heat shock proteins (Hsp72) during fixed-intensity and self-paced exercise in the heat in young, healthy women on oral contraceptives compared with young healthy men.

**Methods:**

Sixteen physically active men and women performed 30 min fixed-intensity cycling at 50% of maximum workload, followed by 30 min of a self-paced time trial (TT) interspersed by 30 s maximal sprint at 9, 19 and 29 min respectively. Trials were undertaken in cool (20°C; 48±3% relative humidity (RH)) and warm (32°C; 66±2% RH) ambient conditions. Core (*T*_c_) and skin temperature, heart rate (HR) and subjective responses were measured before, during and post exercise.

**Results:**

The distance, mean and peak power output, mean and peak speed during the self-paced time trial showed no difference between the ambient temperatures for men and women. Hsp72 in females was higher than males at all sample points at both 20°C and 32°C, except for pre-exercise at 20°C (*p*< 0.04). Women also attained a higher *T*_c_ than men at the end of the TT in the heat (38.5°C v 37.9°C for women and men, respectively; *p*<0.03), higher mean HR and perceived exertion.

**Conclusion:**

This study indicates that females who use oral contraceptives (OC) had higher levels of Hsp72 than males when tested under the same environmental conditions.

Heat shock proteins (Hsps) are a group of highly conserved proteins which are present in the cells of all living organisms and they respond to a variety of physiological and environmental stressors. ^[1]^ Hsps serve as molecular chaperones and accelerate cellular repair from heat stress, ischemia and endotoxic shock. ^[2]^ Temperature is an important stimulus which contributes to the reported exercise-induced increase in Hsps.^[3]^ Heat stress during exercise presents both thermal and sympathetic challenges, with increases in physiological strain compared with moderate temperature conditions ^[4]^, although it appears that the Hsp response is exercise duration and intensity dependent.^[5]^ Further, the exercise stress response appears to be mediated by sex hormones ^[6]^, although the mechanism is not clearly understood. In female rats, exercise-induced elevations in Hsp70 is negatively correlated with circulating oestrogen immediately before exercise.^[7]^ Rats treated with oestrogen regulate at a lower core temperature (*T*_c_) during heat exposure with increased evaporated water loss at all levels of *T*_c_, and with a decreased *T*_c_ threshold at the onset of saliva spreading.^[8]^

Progesterone is known to elicit a thermogenic effect, raising female *T*_c_ in the luteal phase (LP) of the menstrual cycle by ~0.5°C. ^[9–11]^ For women of reproductive age, menstruation is divided into two main phases, each governed by synthesis and the release of pituitary and ovarian hormones. The follicular phase (FP) occurs during approximately the first 14 d of a 28 d cycle and is characterised initially by low levels of oestrogen, progesterone, follicle-stimulating hormone (FSH) and lutenising hormone (LH).^[7]^ As FP progresses from 1–14 d, circulating levels of LH and oestrogen gradually increase, stimulating the release of a mature oocyte from an ovarian follicle, termed ovulation, after which the luteal phase begins. ^[12]^ If fertilisation does not occur during this second phase when progesterone concentrations are highest for 7–10 days (d), the progesterone and oestrogen levels begin to decline, providing the stimulus for uterine shedding (menstruation) and the beginning of the cycle from Day One. ^[12]^ The monophasic oral contraceptive pill (OC) is a formulation of exogenous hormones providing low doses of progesterone and oestrogen for 21 d (active phase) and a ‘withdrawal’ phase of seven d of sugar pills, which is the inactive phase. ^[13] [14]^ There is no consensus and little evidence exists regarding the combined effects of OC on exercise performance during heat stress and the production of cellular chaperones. Exercise duration and intensity, combined with an elevated *T*_c_, are likely precursors for the appearance of cellular chaperones. We are unaware of any studies examining the combined effects of OC use, prolonged self-paced exercise and the appearance of circulating Hsp.

Therefore the aim of this study was to determine the heat shock protein response (Hsp72) during fixed intensity and self-paced exercise in the heat in young, healthy women on oral contraceptives compared with young healthy men.

## Methods

### Participants and study design

Sixteen healthy and physically active participants (males n=8; mean±SD; age 22.1±5.3 yrs; mass 74.2±5.1 kg; height 1.78±0.03 m; peak oxygen consumption (*V*O_2peak_) 3.9±0.7 l·min^−1^) and females (n=8; mean±SD; age 20.9±2.9 yrs; mass 67.9±12.4 kg; height 1.63±0.06 m; *V*O_2peak_ 2.3±0.42 l·min^−1^) gave written informed consent to participate in the study, which was approved by the Institutional Ethics in Human Research Committee, Charles Stuart University. Before starting the experimental trials, all participants were required to make an appointment with a physician and be cleared as healthy and able to undertake the experimental protocol. Participants were non-smokers, physically active for at least one-h, three times per week, not acclimatised to exercising in the heat and free from injury. Female participants had been taking a monophasic OC (Levlen 28; 30 μg ethinyloestradiol and 150 mg levonorgestrel and Brenda-35 ED; 35 μg ethinyloestradiol and 2 mg cyproterone acetate) for at least six months prior to testing. All males were free of pharmacological intervention. Each participant completed two tests, one each in a moderate (20°C) and warm (32°C) environment respectively. The females were tested during the active pill phase of the OC cycle to control for potential effects from fluctuations of natural sex hormones.

Participants reported to the laboratory on three separate occasions to control for diurnal variations. The first visit was for familiarisation with the equipment and study conditions and to obtain a measure of *V*O_2peak_. The remaining laboratory sessions were identical and conducted in either a warm ambient temperature of 32°C or a moderate ambient temperature of 20°C, which were completed in a randomised/counterbalanced order to control for any changes in reproductive hormones throughout testing. Women were tested during the active pill phase to ensure that they received a similar amount of synthetic oestrogen and progesterone derivatives each day. As such, women taking OC do not experience the follicular and luteal phases of the natural menstrual cycle due to ‘active’ hormones action ^[15]^. Testing sessions were conducted on Day eight with exercise in 20°C (F20) and Day 18 of taking hormone pills with exercise in 32°C (F32). These days were chosen for testing as Day eight represents the part of the menstrual cycle when the body temperature will be normal, whereas Day 18 would normally be when the body temperature is higher but is normalised due to the active phase of OC.^[16, 17]^ Males were tested in both moderate and warm conditions separated by at least seven days.

Participants refrained from exercise, alcohol or caffeine consumption for 12 h preceding testing. A 24 h food diary was maintained for the day prior to the first test so that individuals could follow similar eating patterns immediately prior to the remaining tests. Nude mass was measured to the nearest 10 g after avoiding food and a venous cannula was introduced into a superficial forearm vein for repeated blood sampling. Thermistors were attached to the skin.

### Peak testing and performance protocol

The *V*O_2peak_ test was conducted as previously described. ^[16]^ Four hours prior to reporting to the laboratory, participants ingested a telemetry pill (Vital Sense®, Mini Mitter Company Inc., USA) for the measurement of *T*_c_, recorded at five min intervals. Skin thermistors were fastened to four sites as previously described and a mean skin temperature (*T*_s_) ^[18]^ was calculated at five min intervals. Cycle testing was performed with the same apparatus used during the *V*O_2peak_ test, with data recorded by means of Fortius Software for Cosmos Ergometer (v1.29, Tacx bv, Netherlands).

To distinguish between physiological responses which occur during fixed intensity versus self-paced exercise, the endurance test was split into 2 × 30 min sections ^[19][20]^; 30 min of fixed intensity followed by 30 min of self-paced exercise. Participants commenced the 30 min fixed-intensity cycling at 50% workload maximum (W_max_) calculated from the *V*O_2peak_ test programmed at the beginning of each trial using Fortius Software to ensure that fixed intensity was maintained and with gear settings kept constant throughout. Subsequently, a three min rest was provided in the climate chamber to allow for blood collection. Immediately following the rest period, a 30 min self-paced time trial (TT) commenced. During the fixed intensity and TT a series of ‘all out’ 30 s sprints were completed at 9, 19 and 29 min marks respectively. The participant was instructed to cycle as far as possible in the 30 min TT but was permitted to change gears as desired. No feedback was provided to the participant during any part of the trial other than a countdown to the next sprint given at two min, 30 s and 10 s intervals respectively, with strong verbal encouragement provided. Upon the completion of the TT, blood samples were collected before the participant exited the climate chamber. A final nude body mass was recorded to estimate total body sweating. Distance covered in km, average and peak cadence in rpm, average and peak speed in km.h^−1^ and average and peak power (W) were recorded by means of the Tacx software at five min intervals throughout the cycling protocol.

### Physiological and subjective measures

A rating of perceived exertion (RPE; 1–10 Scale) ^[22]^ and thermal sensation on a scale from one representing ‘cold’ to seven representing ‘hot’ ^[23]^ were recorded at five min intervals from the start to completion of the final sprint. Heart rate was continuously monitored and recorded at five min intervals (FS1; Polar Electro Oy, Kempele, Finland). Feedback was not available to participants, with the receiver obscured from the participant’s view.

Blood samples were drawn from a superficial forearm vein immediately following the cannula set-up, following the fixed intensity protocol and upon completion of the TT. Catheter patency was maintained by flushing with 0.9% sodium chloride (Pfizer, Australia) after each blood draw and ~ at five min intervals. Blood samples were divided into pre-cooled serum separator tubes for the determination of heat shock protein 72 (Hsp72). To examine the potential effect of exercise and heat stress on hormonal stress response ^[24]^, a sample was allocated to a pre-cooled K_3_EDTA tube for determination of cortisol (Vacutainer, S-Monovette, Sarstedt, Germany) To evaluate the metabolic intensity of exercise a 0.5 ml aliquot of whole blood was drawn into a syringe for determination of lactate (La^−^) (ABL800 Flex Radiometer, Copenhagen). Collected blood was centrifuged at 4 500 rpm in a refrigerated centrifuge for 10 min. Separated plasma was placed into one ml aliquots and frozen at −80°C until further analysis.

Before the analysis of Hsp72, the serum was thawed to room temperature and mixed gently via inversion. Duplicate plasma Hsp72 concentrations were measured using an ELISA kit (Anti-Human Hsp70 (total) ELISA Kit, Assay Designs Inc., Ann Arbor, MI, USA), with detection limits of 31.25 ng.ml^−1^. To avoid inter-assay variations, all samples were assayed in the same assay run. Serum protein concentrations were not corrected for plasma volume shifts, thus all statistical analyses were performed on actual measured circulating concentrations. ^[14]^

### Statistical analysis

A priori power calculations were conducted using G*Power (G*Power 3.1.2, Franz Faul, Germany), which indicated eight participants were needed. Repeated measures ANOVAs were used to determine differences between environmental and OC conditions in cycling performance or biochemical markers. When interactions or main effects achieved statistical significance, Tukey’s HSD post hoc test was used to identify differences between means. Statistical significance was set at *p*<0.05. Data are reported as mean±SD.

## Results

### Exercise performance

Table 1 provides the various parameters measured during both fixed intensity and self-paced trials for each condition. For the fixed intensity bout, distance cycled, mean and peak power output and the mean and maximal speed did not differ for either gender or between ambient conditions. Cycling speed during the sprints was significantly faster compared to the mean speed by ~27 km·h^−1^ in both F20 and F32 (*p*<0.001) and ~33 km·h^−1^ in both M20 and M32 (*p*<0.001), respectively. For the self-paced bout, distance cycled, mean and peak power output and mean and maximal speed did not differ between ambient temperatures for the respective genders. However, maximal speed was about seven km·h^−1^faster in F32 compared with F20 (*p*<0.007). In addition, maximal speed and peak power outputs were significantly greater than the mean speed and power outputs in all conditions *(p<*0.05).

### Thermoregulatory responses

**Fig. 1:**
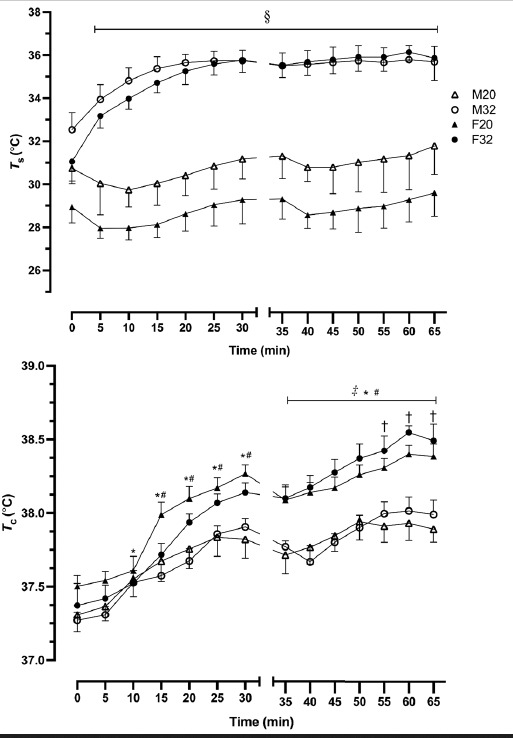
Mean skin temperature (T_s_; top panel) and core temperature (T_c_; bottom panel) response during fixed-intensity (0 - 30 min) and self-paced time trial performance (35 – 65 min). F, female (n=8); M, male (n=8); 20 and 32 are 20°C and 32°C ambient temperatures, respectively. § indicates p < 0.05 compared with F20 and M20. ‡ indicates p < 0.05 compared with F20 and M20 and between M32 and F32 values; * indicates p < 0.05 from pre-exercise in M20 and M32; # indicates p < 0.05 from pre-exercise in F20 and F32; † indicates p < 0.05 between M20 and F20.

The *T*_c_ responses to exercise are shown in Figure 1. In the fixed intensity section, *T*_c_ was significantly elevated from baseline at 10 and 15 min of exercise for males and females respectively (*p*<0.05). Time trials in both conditions did not result in a significant change in *T*_c_ between males and females, although a trend from 20 min onwards for a higher *T*_c_ in F32 compared with M32 and at the completion of cycling in 20°C between males and females was evident. Self-paced cycling at 32°C resulted in significantly higher *T*_c_ in females compared with males at all time points from the commencement of the self-paced cycling (*p*<0.03). In 20°C, females exercised with higher *T*_c_ than males at a 20 min period until the completion of exercise (*p*<0.05).

During the cycling exercise, *T*_s_ was significantly elevated from baseline by 10 min in all conditions, until the completion of exercise (*p*<0.05). The *T*_s_ at 32°C was higher than that reached during 20°C, for both males and females *(p<*0.05). At 32°C, males reached 35.7°C and females reached 35.9°C, whereas at 20°C males reached 31.8°C and females 29.6°C.

### Heart rate and subjective responses

Table 2 lists the mean HR and RPE responses for each of the low intensity and sprint sections of the trials in each ambient condition. Heart rate was significantly elevated from baseline, ~93 bpm and ~86 bpm for males and females respectively, in all conditions (*p*<0.05), and all sprint values were higher than mean values. The mean HR during fixed intensity cycling were similar for males and females but were higher during the non-sprint periods. Throughout the time trial, mean HR for males and females, compared with lower intensity periods was higher. The HR responses were not statistically significant between males and females in either condition.

RPE responses followed that of HR, in that RPE significantly increased from baseline and was elevated immediately following the sprints, and then significantly decreased in the lower intensity period (*p*<0.05). Mean RPE was significantly higher for the sprints in each trial compared with the low intensity efforts. Mean RPE was higher at the 32°C condition compared with that at the20°C for both males and females. However, there were no differences between males and females in the same ambient conditions. Thermal sensation was significantly higher in the 32°C trial compared with the 20°C trial at all time points, for both genders (*p* < 0.05). However, there were no differences between males and females in the same ambient conditions.

### Hsp responses

**Fig. 2:**
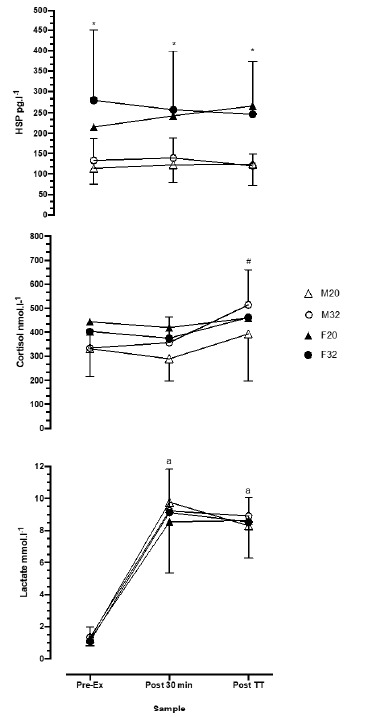
Heat shock protein (HSP; top panel), cortisol (middle panel) and lactate (bottom panel) responses pre-exercise (Pre-Ex), end of the 30 min fixed intensity cycle (Post-30 min) and at the end of the 30 min time trial (Post TT). F, female (n=8); M, male (n=8); 20 and 32 are 20°C and 32°C ambient temperatures, respectively. * indicates p < 0.04 from pre-exercise in M20 and M32; # indicates p < 0.04 for M32 at Post TT compared with Pre-Ex; a indicates p < 0.01 vs Pre-Ex in both genders across all conditions.

Figure 2 shows the Hsp72 response at the three time points of collection. Hsp in females was higher than males at all corresponding sample points for both 20°C and 32°C (*p*< 0.05) conditions. There were no differences within each condition for males or females. For cortisol, M32 increased significantly by the completion of the self-paced exercise, compared with the pre-exercise and fixed-intensity cycling (334, 357 and 514 nmol·l^−1^, for pre-exercise, fixed-intensity and self-paced exercise, respectively; *p*<0.04). Lactate samples in all conditions increased from pre-exercise and are shown in Figure 2. Lactate was not significantly different among genders, ambient temperature or fixed-intensity versus self-paced cycling exercise. Both genders reached ~9.1 nmol·l^−1^ at the completion of fixed-intensity cycling and ~8.8 nmol·l^−1^ at the completion of the self-paced cycling.

## Discussion

To the best of our knowledge, this is the first study to report the Hsp response during cycling exercise performed in different ambient conditions in females using OC in comparison to untreated males. We observed that females exercising in both moderate and warm ambient conditions had a significantly higher Hsp72 response compared with the men exercising in the same environments. The reason for this elevated response is not entirely clear; however, it is plausible that this response may be attributed to the elevated heat strain experienced by females as shown in Figure 1. Females exercised at a higher *T*_c_ than males, although this difference was not significant until 30 min of F20 fixed-intensity and from the beginning of the TT in F32. The difference in *T*_c_ value was approximately 0.5°C at the completion of both cycling bouts, which aligns with reports that females in the luteal phase of the menstrual cycle experience an elevation in *T*_c_ of 0.3 – 0.5°C, due to elevated circulating progesterone. ^[10]^ Previously, it has been shown in animal studies that body temperature elevation during exercise is important for the induction of exercise increases of Hsp72 ^[3]^. In human studies it appears that men and women differ in their cellular stress response, where men up-regulated their Hsp72 response after a single bout of exercise in the heat, persisting for 12 days which confers cellular thermotolerance ^[6]^. However, this upregulation appears highly dependent on the level of exercise-induced hyperthermia that is achieved after one bout of exercise, and, as shown in the present study, this was lower for males than for females.

An additional factor which may explain the difference in the Hsp72 response is that the women in the present study did not experience the fluctuations of the natural menstrual cycle as they were ingesting a monophasic OC formulation. This provides a steady, albeit low dose of oestrogen and progesterone over 21 days before the withdrawal phase. Since oestrogen might mediate the stress response by stabilising cell membranes, OC use may interfere with this mechanism by reducing the natural oestrogen levels; thereby augmenting the need for upregulating Hsp72 during acute stress. ^[6]^ Presumably, women on OC are free of the day-to-day variations in body temperature which is characteristic of natural ovulatory cycles. However, synthetic hormones interfere with thermoregulation, elevating the body temperature consistently over 24 h, to the same extent as it is in ovulating women in the LP. ^[11, 25]^

Although not statistically different, during fixed-intensity cycling the distance covered in 30 min was 7% further for males compared with females. The fixed-intensity power output was ~25% higher for males and they were also able to produce higher mean and maximal speeds (~8% and ~13% increases, respectively). Although there were no statistically significant differences in the self-paced exercise, males cycled ~16% further than females (13.0 km compared with 10.9 km) and produced significantly higher mean (~32% increase) and peak (~42% increase) power output and mean (~11% increase) and peak (~21% increase) speed in both conditions. Despite the higher intensity that males were able to maintain during the cycling exercise, there was no difference in thermal sensation and in fact, females sustained higher HR, RPE and *T*_c_ than their male counterparts.

The increased *T*_c_ in females in this study could potentially explain the higher values of Hsp72 compared to males. Hsp72 for female participants was higher at rest, and significantly elevated at the completion of the fixed-intensity cycling and at the completion of the self-paced cycling compared to males (~49% higher at all time points). There was no statistical difference between ambient temperatures, although in the F32 condition, the Hsp72 concentration was higher than in the F20 condition. However, the Hsp72 response was not reflective of circulating cortisol as only M32 produced significant increases during the self-paced cycling. Although all other conditions demonstrated elevations in cortisol, these differences were not significant. Thus, it is likely that the elevation in Hsp72 is unrelated to the stress response to exercising in the heat.

It is also possible that changes in cortisol and Hsp72 were not completely developed over the course of the study’s exercise bout, as it is purported that the cellular chaperone response is duration and intensity-dependent. ^[11]^ Thus, it is possible that the cycling exercise used here was insufficient to induce high levels of cellular stress. Further, it is possible that the self-paced exercise allowed the participants to adjust their efforts to preserve homeostasis as much as possible. ^[26, 27]^ Further explanations about the differences in HSP response in the present study include differences in muscle mass and body composition, surface area to volume ratio, sweat rate and biomechanical efficiency, none of which were examined here. Prospective research could examine these differences in conjunction with longer, possibly more intense cycling exercise.

Finally, there are several limitations which limit the interpretation of our results. First, we did not directly compare normally ovulating females not taking OC with either females taking OC or compared to males. We limited our study to the role of OC use within females over the regular menstrual cycle. As such, the Hsp72 response to exercise in females not taking OC is unknown. Therefore, we cannot conclude that taking OC has any impact on the Hsp72 response to exercise. All that we can conclude is that the Hsp72 response in females taking OC was higher in both warm and moderate conditions compared with males exercising under similar conditions. We also cannot account for individual diurnal variation in the timing/phases of the menstrual cycle, thus we may not have completed testing precisely on the appropriate cycle day for each participant when *T*_c_ might have been highest if they were not taking OC.

## Conclusion

This study indicates that females who use an OC had higher levels of Hsp72 than males when tested under the same environmental conditions. The definitive reasons for this are currently unclear although females achieved a higher *T*_c_ at the completion of the self-paced cycling, which, combined with the low dose of oestrogen and progesterone from the OC pill may have mediated the stress response by stabilising the cell membranes. Further research is required to establish the mechanisms involved in the Hsp72 response to self-paced cycling in the heat and whether there are consistent differences between males and females.

## Figures and Tables

**Table 1:** Fixed-intensity and self-paced cycling performance measures

	Fixed intensity				Time trial			
	F20	F32	M20	M32	F20	F32	M20	M32
**Total distance (km)**	15.7 ± 3.7	15.9 ± 3.1	16.8 ± 3.2	17.0 ± 2.9	10.8 ± 1.2	10.9 ± 1.1	13.0 ± 1.8	13.1 ± 2.6
**Mean power output (W)**	116 ± 20	116 ± 20	155 ± 23	155 ± 23	126 ± 16	128 ± 15	189 ± 48	187 ± 65
**Peak power output sprints (W)**	116 ± 20	116 ± 20	155 ± 23	155 ± 23	350 ± 98#	365 ± 65#	611 ± 76#	609 ± 113#
**Mean speed (km.h** ^**−1**^ **)**	30.9 ± 7.2	31.1 ± 6.0	33.8 ± 6.0	32.9 ± 5.8	21.2 ± 2.4	21.4 ± 1.9	24.5 ± 4.0	22.9 ± 5.1
**Max speed sprints (km.h** ^**−1**^ **)**	57.7 ± 3.4*	57.8 ± 6.0*	66.3 ± 1.0*	66.4 ± 1.4*	34.2 ± 3.5*	40.9 ± 7.0*^	46.8 ± 5.4*	49.5 ± 7.9*

Data expressed as mean ± SD. F, female (n=8); M, male (n=8); 20 and 32 are 20°C and 32°C ambient temperatures, respectively.

* indicates p<0.05 where maximal speed is higher than mean speed in all conditions.

# indicates p<0.05 where peak power output is higher than mean power output in all conditions.

^ indicates p<0.05 where peak speed is higher in F32 compared with F20.

**Table 2:** Heart rate (HR) and rating of perceived exertion (RPE) during the low intensity effort and sprints in fixed intensity and self-paced trials.

	Intensity	F20	F32	M20	M32
**Fixed-intensity**	**Low**				
	HR (bpm)	140 ± 6	140 ± 11	130 ± 9	132 ± 10
	RPE (au)	3.7 ± 1.3	3.6 ± 1.6	3.1 ± 1.1	3.3 ± 1.2

	**Sprints**				
	HR (bpm)	171 ± 2*	173 ± 10*	172 ± 1*	173 ± 6*
	RPE (au)	5.3 ± 1.7*	5.6 ± 2.1*	6.0 ± 0.6*	6.1 ± 1.1*

**Self-paced**	**Low**				
	HR (bpm)	150 ± 3	163 ± 1	143 ± 2	148 ± 1
	RPE (au)	5.7 ± 1.6#	6.7 ± 1.8	4.0 ± 0.3	5.0 ± 0.8§

	**Sprints**				
	HR (bpm)	175 ± 3*	179 ± 1*	174 ± 1*	175 ± 1*
	RPE (au)	7.3 ± 1.2*	8.1 ± 1.5*§	7.1 ± 0.4*	7.8 ± 0.5*

Data expressed as mean ± SD. F, female (n=8); M, male (n=8); 20 and 32 are 20°C and 32°C ambient temperatures, respectively; HR, heart rate; RPE, rating of perceived exertion; bpm, beats per minute; au, arbitrary unit. Low and Sprints are the intensities during the fixed intensity and self-paced sections of the trials in each ambient temperature.

* indicates p < 0.05 increase from low to high intensity efforts in all conditions;

§ indicates p < 0.05 between ambient conditions for each gender;

# indicates p < 0.05 increase compared with the male mean value in same ambient condition.
